# Association of ionizing radiation dose from common medical diagnostic procedures and lymphoma risk in the Epilymph case-control study

**DOI:** 10.1371/journal.pone.0235658

**Published:** 2020-07-10

**Authors:** Elisa Pasqual, Michelle C. Turner, Esther Gracia-Lavedan, Delphine Casabonne, Yolanda Benavente, Isabelle Thierry Chef, Marc Maynadié, Pierluigi Cocco, Anthony Staines, Lenka Foretova, Alexandra Nieters, Paolo Boffetta, Paul Brennan, Elisabeth Cardis, Silvia de Sanjose

**Affiliations:** 1 Barcelona Institute for Global Health (ISGlobal), Barcelona, Spain; 2 Universitat Pompeu Fabra (UPF), Barcelona, Spain; 3 CIBER Epidemiología y Salud Pública (CIBERESP), Madrid, Spain; 4 McLaughlin Centre for Population Health Risk Assessment, University of Ottawa, Ottawa, Canada; 5 Unit of Infections and Cancer, Cancer Epidemiology Research Programme, IDIBELL, Catalan Institute of Oncology, Barcelona, Spain; 6 Registre des Hémopathies Malignes de Côte d’Or INSERM U 1231, Université de Bourgogne Franche-Comté et CHU Dijon-Bourgogne, Dijon, France; 7 Department of Public Health, Clinical and Molecular Medicine, Occupational Health Section, University of Cagliari, Cagliari, Italy; 8 School of Nursing and Human Science, Dublin City University, Glasnevin, Dublin, Ireland; 9 Cancer Epidemiology and Genetics, Masaryk Memorial Cancer Institute and MF MU, Brno, Czech Republic; 10 Centre of Chronic Immunodeficiency, Molecular Epidemiology, University Medical Center Freiburg, Freiburg, Germany; 11 Tisch Cancer Institute and Institute for Translational Epidemiology, Icahn School of Medicine at Mount Sinai, New York, NY, United States of America; 12 IARC, International Agency for Research on Cancer, Lyon, France; Northwestern University Feinberg School of Medicine, UNITED STATES

## Abstract

Medical diagnostic X-rays are an important source of ionizing radiation (IR) exposure in the general population; however, it is unclear if the resulting low patient doses increase lymphoma risk. We examined the association between lifetime medical diagnostic X-ray dose and lymphoma risk, taking into account potential confounding factors, including medical history. The international Epilymph study (conducted in the Czech-Republic, France, Germany, Ireland, Italy, and Spain) collected self-reported information on common diagnostic X-ray procedures from 2,362 lymphoma cases and 2,465 frequency-matched (age, sex, country) controls. Individual lifetime cumulative bone marrow (BM) dose was estimated using time period-based dose estimates for different procedures and body parts. The association between categories of BM dose and lymphoma risk was examined using unconditional logistic regression models adjusting for matching factors, socioeconomic variables, and the presence of underlying medical conditions (atopic, autoimmune, infectious diseases, osteoarthritis, having had a sick childhood, and family history of lymphoma) as potential confounders of the association. Cumulative BM dose was low (median 2.25 mGy) and was not positively associated with lymphoma risk. Odds ratios (ORs) were consistently less than 1.0 in all dose categories compared to the reference category (less than 1 mGy). Results were similar after adjustment for potential confounding factors, when using different exposure scenarios, and in analyses by lymphoma subtype and by type of control (hospital-, population-based). Overall no increased risk of lymphoma was observed. The reduced ORs may be related to unmeasured confounding or other sources of systematic bias.We found little evidence that chronic medical conditions confound lymphoma risk and medical radiation associations.

## Introduction

The use of ionizing radiation (IR) in medicine has significantly improved patient care. However, it has also resulted in a large increase in IR exposure to the general population [1–3], thus raising concerns in the public health and radiological protection communities. Medical diagnostic procedures typically deliver low to moderate IR doses. Estimating risk of lymphoma at these low dose levels represents a challenge for epidemiology. Lymphomas are mainly classified into Hodgkin Lymphomas (HL) and Non Hodgkin Lymphomas (NHL), and are initiated by a mutation in a lymphoid stem cell. Lymphoid cells originate in the bone marrow (BM), a radiosensitive organ; at present, it is unclear whether IR increases the risk of lymphoma risk, particularly at low doses [4–7].

Table 1 summarizes results of major studies of lymphoma risk and low-dose IR exposure. In studies of atomic bomb survivors, the incidence of malignant lymphoma and mortality of NHL were both positively associated with radiation exposure in men, but not in women [8, 9]. In the largest combined analysis of nuclear workers studies, a non-statistically significant increased risk was found for both HL and NHL mortality [10]. An increase in NHL incidence was also observed in Chernobyl liquidators [11]. Results of previous studies of medically-exposed subjects are mixed and unclear. There was no positive association of NHL and medical diagnostic radiation exposure in two studies in the US and Australia [12, 13]. Similarly, no increased HL risk from pediatric Computer Tomography (CT)-scan exposure [14] was found in the UK, though an Australian CT-cohort reported a significant increase in risk when comparing subjects exposed to CT-scans to those unexposed [13].

**Table 1:** Summary of previous studies evaluating lymphoma risk after ionizing radiation exposure.

Reference	Study design and description of the population	Exposure details	Results	Conclusions
**Outcome = Malignant lymphoma (combining NHL and HL)**				
[9]	Mortality—**A-bomb survivors cohort study**–follow-up to 2003 (N = 120321; 284 lymphoma cases)	Bone marrow dose, Range 0 to 4Gy	ERR/10 mGy^a^ 0.0016, 95% CI (-0.0013; 0.027)	No increased risk of lymphoma mortality overall. Increase in men but not in women, possibly reflecting a disparity in lymphoma subtypes by gender.
			ERR/10 mGy^a^ 0.007, 95% CI (0.0008; 0.017) in males	
			ERR/10 mGy^a^ -0.0018, 95%CI (-0.0021; 0.0024) in females	
[15]	Mortality—**A-bomb survivors cohort study**–follow-up to 2000 male aged 16–64 at time of bombing (N = 20940; 90 lymphoma cases)	Radiation dose absorbed by the colon^b^, Range 0 to 4 Sv	ERR/ 10 mSv^c^ 0.0079, 90% CI (0.001; 0.0188), 5 year lag	Evidence of an increased risk
	Mortality—Savannah River Site (SRS) US nuclear weapons worker cohort study (N = 15264; 56 lymphoma cases)	**Occupational;** Cumulative whole body radiation, 0–0.5 Sv	ERR/10 mSv^c^ 0.0699 90%CI (0.0096; 0.1839)	Evidence of an increased risk
[16]	Incidence—Cohort of children/adolescent undergoing a cardiac catheterization procedure (N = 11270; 22 lymphoma cases)	**Medical procedures (cardiac catheterization and CT-scan);** cumulative bone marrow radiation: median 3.1; p25 1.3 and p75 9.3 mGy	SIR for lymphoma 9.15 95% CI (5.66–13.97) with 2 years lag period	Lymphoma rate among patients receiving cardiac catheterization is high compared with general population. It is likely that transplant (immunodepressive medication) is the most likely causal factor.
**Outcome = Non Hodgkin Lymphoma**				
[8]	Incidence–**A-bomb survivors** cohort study–follow-up 1950–2001 [N_ = 113,011; 402 NHL]	Bone marrow dose, Range 0 to 4 Sv	ERR/10 mGy 0.0046, 95% CI (-0.0008; 0.0129) in males	Weak support for an association between radiation and NHL in men
			ERR/10 mGy 0.0002, 95% CI (-0.0044; 0.0064) in females	
[10]	Mortality—INWORKS study, nuclear industry workers from US, France, UK [N = 113,011 and 710 NHL]	**Occupational**; cumulative lifetime bone marrow dose, Mean(range), 15.9 (0–1217.5) mGy	ERR/10 mGy 0.0047, 90%CI (-0.0076 to 0.02)	Positive but non significant association.
[11]	Case-control study of hematological malignancies (20 NHL / 80 controls) in Chernobyl liquidators	Bone marrow doses from work as a **clean-up worker**. 78% < 50 mGy; 14% > = 100 mGy.	ERR/10 mGy 0.281, 95% CI (0.009; 2.43)	Increase risk of NHL after exposure to IR
[12]	Lymphoma case-control study (318 cases, 449 controls)—US	**Medical diagnostic radiation**; cumulative lifetime bone marrow dose (based on literature review). Dose not reported. Category cut point are 1, 2,3 and 4 mGy	RR for the exposed versus unexposed 0.99, 95% CI (0.6; 1.6); RR of the highest exposure category versus lowest 1.4 (CI not reported)	No statistically significant association found
**Outcome = Hodgkin Lymphoma**				
[8]	Incidence–**A-bomb survivors** cohort study follow-up 1950–2001 [N = 113,011; 35 HL]	Bone marrow dose, Range 0 to 4 Sv	ERR/10 mGy 0.002, 95% CI (-0.0103; 0.0263)	No evidence for an increased risk Small number of cases
[10]	Mortality—INWORKS study, nuclear industry workers from US, France, UK [308,297 cohort size; 104 HL cases]	**Occupational;** cumulative bone marrow dose, Mean(range), 15.9 (0–1217.5) mGy	ERR/10 mGy 0.0294, 95% CI (n.e; 0.1149)	Positive but not significant association, Small number of cases
[14]	UK cohort of CT-scan exposed children [178,601 cohort size; 65 HL cases];	**CT-scan exposure;** cumulative bone marrow dose (mean 11 mGy, range 0–689 mGy)	ERR/10 mGy 0.02, 95% CI (-0.16; 0.21), 2 year lag-period, Bone marrow dose	No association, Small number of cases
		**CT-scan exposure;** cumulative lymphoid dose (mean 8mGy, range 0–348 mGy).	ERR/10 mGy 0.28, 95% CI (-0.24; 0.8), 2 year lag period	
[13]	Australia, population based cohort of CT exposed children [10.939.680 cohort size; 228 HL]	**CT-scan exposure;** Exposed (680.211 people) vs Unexposed	IRR 1.15, 95%CI (1.01 to 1.32), 1 year lag-period	Risk increased, No dose estimate. Reverse causation could also explain this result (short lag-period)
**Outcome = Chronic lymphocytic leukemia**				
[8]	Incidence–**A-bomb survivors** cohort study follow-up 1950–2001 [N = 113,011; 12 CLL]	Bone marrow dose, Range 0 to 4 Sv	Statistically significant linear trend (P <0.05)	Suggest a possible increase in CLL, Small number of cases
[10]	Mortality data of INWORKS study, nuclear industry workers from US, France, UK [138 deaths]	**Occupational;** cumulative bone marrow dose Mean(range), 15.9 (0–1217.5) mGy	ERR/10 mGy -0.0106, 90% CI (n.e.; 0.0181)	No association
[17]	**Nested case-control study of Ukraine clean-up workers** of Chernobyl accident (cohort size 110,645; 65 CLL cases)	Bone marrow doses from work as a **clean-up worker**. Mean (SD) of cumulative BM dose 132.3 (342,6) mGy in cases and 81.8 (193,7) mGy in controls	ERR/10 mGy 0.0258, 95% CI (0.0002; 0.0843)	Increased risk of CLL
[11]	Case-control study of hematological malignancies (21 CLL / 80 controls) in Chernobyl liquidators	Bone marrow doses from work as a **clean-up worker**. 78% < 50 mGy; 14% > = 100 mGy.	ERR/10 mGy 0.047, 90% CI (n.e.; 0.76)	Non-significantly increased risk of CLL associated with radiation exposure, Small number of cases
[18]	Nuclear worker study (15-country study; 295,963 workers), mortality analysis (65 CLL deaths)	**Occupational;** cumulative bone marrow dose, Mean 14.7 mSv	RR at 100 mSv compared to 0 mSv: 0.84, 95% CI (0.39, 1.48)	No association. Few CLL deaths
[19]	Nested case-control study of worker of US nuclear weapon facilities (94517 cohort size; 43 deaths and 172 controls)	**Occupational,** lifetime cumulative bone marrow dose + work-related medical X-ray exposure. Median dose in cases was 1.4 mSv	ERR/10mSv^c^ -0.020, 95% CI (n.e. to 0.14). No CLL case above 100 mSv.	Little evidence for an association, Small number of deaths
			ERR/10mSv^c^ excluding subjects with dose above 100 mSv^c^ 0.16, 95% CI (-0.044; 0.83)	
[20]	Case-cohort study in 23,043 uranium miners (53 CLL cases)	**Occupational** radon exposure, (using industry tables of mean annual 222Rn concentrations)	RR for highest exposure category compared to lowest quintile of distribution^d^ 1.98, 95% CI (1.1; 3.59)	Positive association between radon exposure and CLL risk, Small number of cases
[12]	Lymphoma case-control study (207 cases, 449 controls)—US	**Lifetime medical diagnostic exposure**; Exposed versus unexposed	Relative risk 2 year lag period 0.56 (0.3; 1.1 95%CI)	Negative non significant association. Ascertainment bias could be an explanation

ERR: Excessive Relative Risk; RR: Relative risk; IRR: Incidence Rate Ratio; CI: Confidence Interval; SIR: Standardized Incidence Ratio; n.e: not estimable.

^a^In recent atomic bomb survivor studies, the doses are expressed as “weighted doses”, in Gy, rather than equivalent doses in Sv, using a weighting factor of 10 for neutrons, whatever their energy.

^b^Colon dose in atomic bomb survivors reports is usually used for solid cancer risk estimation.

^c^Dose estimated in Sv because the study focused on high energy photon exposure; for comparison purposes 1 Gy of absorbed dose is approximately equal to 1 Sv of equivalent dose.

^d^Highest category corresponds to 110 WLM or more; lowest category to less than 3 WLM. WLM: Working Level Month; a WLM is a unit of exposure defined as an exposure to 1 Working Level (a measure of concentration of alpha particles in air) of radon for one month (170 hours).

Among NHL subtypes, chronic lymphocytic leukemia (CLL) has traditionally been thought not to be radiation-induced, based on lack of evidence of an increased risk in studies of atomic bomb survivors. However, CLL is very rare in Japan and has a long asymptomatic period, hence the study of atomic bomb survivors has low statistical power to detect an increased risk, if any [21]. Increases in the risk of CLL have been noted in a number of nuclear workers studies [19] as well as among Chernobyl clean-up workers (liquidators) [11, 17] and recently also in the atomic bomb survivors incidence follow-up [8]. Thus, the question of whether or not CLL can be induced by radiation is still under debate [19].

A potential concern in estimating lymphoma risk from medical radiation exposure is possible confounding by clinical indication. Medical radiation exposure does not occur at random in the general population; rather, it tends to be related to a person’s underlying medical history. Furthermore, patients with lymphoma tend to share a particular pattern of medical history: they are more likely to suffer from immune deficiencies or infectious diseases (hepatitis B or mononucleosis), and less likely to have an atopic disease [6, 22, 23]. Previous studies of lymphoma risk in relation medical radiation exposure have generally not considered these potential confounding factors [12–14].

Here we examine the association between medical diagnostic IR exposure and lymphoma risk in the multicentre European lymphoma case-control study Epilymph. For this, we estimated cumulative lifetime BM dose from the study participants’ self-reported medical radiological histories. The exposure quantity used here was cumulative absorbed dose to the BM, the quantity generally used in previous studies of lymphoma risk [8, 14]. We then explored the association between lifetime BM dose and lymphoma risk taking into account the potential confounding effect of medical history variables.

## Materials and methods

### Study design

Epilymph is a case-control study of incident lymphoma conducted between 1998 and 2004 in the Czech-Republic, France, Germany, Ireland, Italy, and Spain. Cases were recruited as soon as possible after the diagnosis to minimize the possibility of any survival bias. Controls were frequency matched by 5-year age groups, sex and country of residence and randomly selected from population registries in Italy and Germany (population-based recruitment) and among patients admitted to the same hospital as cases in the remaining countries (hospital-based recruitment). The proportion of hospital-controls who had the same diagnosis for hospital admission was kept lower than 10%. Participation consisted of an in-person interview on the subject`s socioeconomic status (SES), lifestyle factors and medical history, including medical radiological history. Interviews were conducted by trained personnel using a structured questionnaire. The detailed methods have been published elsewhere [24, 25].

### Exposure estimation

Regarding medical radiological history, the interviewer presented a list of common radio-diagnostic examinations: conventional X-ray to the thorax, abdomen (with and without contrast), kidney, bones and face; dental X-ray; gammagraphy (not collected in Germany), and Computerized Tomography (CT) scans. Participants were asked to report the total number of procedures they underwent and the age at first and last procedure for each type of examination. The precise body part was not asked for bone X-rays, CT-scans or gammagraphy. An additional question about any additional examinations was asked, with the answer recorded as free text. The list of questions asked can be found in S1 File.

We excluded examinations taken during the two years prior to the date of diagnosis/reference date to allow for an appropriate latency period between exposure and diagnosis as well as to exclude any procedure possibly related to the diagnosis of the cases. As only age at first and last procedures were available, we assumed a uniform distribution of examinations between the two reported ages, excluding those that fell within the 2-year exclusion period. A sensitivity analysis using a 5 year exclusion period was also performed. We excluded CT-scans reported before the year 1980 (44 CT-scans reported by 42 subjects) since CT-scans were not routinely performed until the late 1970´s-early 1980’s [26, 27].

Since there were no data on the specific dosimetry protocols followed for each procedure and body part, we based our organ dose estimations on typical protocols used in different decades, using published estimates of organ doses. This approach has been used in other similar studies [12, 13, 28]. For conventional X-rays, Melo [29] provided a dose value for each 10-year period from 1930 to 2010. For CT-scans, Kim et al. [30] provided a dose value by sex for two different time periods (pre- and post-2001) and by year of age (0–22 years). We subsequently calculated a mean dose value for the following age groups: 0–5; 6–10; 11–15; 16–20; and >20 years. Since no body part specification was available for CT-scans or bone X-rays, we attributed to each body part a weighted average of doses to different anatomical regions, according to the frequency of examinations of these regions in the general population [3, 31, 32]. A look-up table was derived to assign doses by type of procedure, body part, and time period (see S1 and S2 Tables).

For the cumulative dose calculation, we excluded 169 gammagraphy procedures (reported by 107 subjects) because of lack of information on the procedure in the study questionnaire, and no information was collected in Germany. We also excluded 9,125 dental X-ray procedures (reported by 2,296 subjects) since the fraction of BM in the mandible is close to 0 [33]. Indeed, the dose to the bone marrow from one intraoral dental X-ray is estimated to range from 10^−3^ to 10^−5^ mGy, depending on the time period [34]. An additional 52 examinations reported in free text by 51 subjects were also excluded as they were too poorly defined to classify.

Cumulative lifetime BM dose was calculated summing doses from all procedures up to the exclusion period. As only age at first and last procedures was available, we attributed: a) to the first and last procedures, the doses in the look-up table corresponding to the time periods and type of procedures. Since there was no data on the precise time period for the remaining *“n-2*” examinations, we used three different scenarios for attributing dose values, as follows: a) the mean of the dose over the entire time period between first and last exposure for that type of procedure (main analysis); b) the dose corresponding to the time period of the first procedure; c) the dose corresponding to the time period of the last procedure. Therefore, for each participant and examination we estimated three possible dose values. Sensitivity analyses using the “b” and “c” scenarios were conducted to compare results under the alternative exposure assumptions. Total cumulative BM dose was then estimated for each scenario by summing across all examination types (S2 File). Cumulative BM dose was categorized into 4 categories, with cut points chosen *a priori*: 1, 5 and 15 mGy (which approximately correspond to the 25^th^, 75^th^ and 95^th^ percentiles of the dose distribution in the exposed controls).

### Outcome definition

For all cases, diagnosis was histologically confirmed and classified according to the World Health Organization (WHO) classification of neoplastic diseases revision 3 [35]. For the main analysis we considered all lymphomas together. Additional analyses were conducted by subtype: NHL, HL and CLL. In addition an analysis with all lymphomas excluding CLL was performed for consistency with previous radiation epidemiology papers [10].

### Definition of covariates

Data on personal and family medical history were collected as part of the study interview by asking if the subject or one of his/her family members had ever suffered from any of a number of medical conditions and, if so, the age at onset. The conditions were grouped as follows: a) family history of lymphoma; b) any autoimmune disease (arthritis, lupus, celiac disease and rheumatic fever); c) any atopic disease (asthma, eczema, allergy, urticaria and sinusitis); d) any infectious disease (mononucleosis, tuberculosis, hepatitis, brucellosis and typhoid fever); e) osteoarthritis; f) cardiovascular disease (diabetes, hypertension). A measure of global health status in childhood was also captured by asking the participants about their level of health during childhood using five questions (having been more sick/absent from school comparing with schoolmates; taking more medicines/antibiotics comparing with siblings; having been a very healthy child) [25]. Two measures of Socio-Economic Status (SES) were also captured: a) years of school attendance (categorized using cut points of: 10, 13 years); and b) socio-economic status based on the Standard International Occupational Prestige Scale (SIOPS) [36] for the longest held occupation (categorized using quartiles of the SIOPS score distribution among controls). The correlation between the two SES measures was low (Pearson r = 0.39).

### Statistical analysis

We examined associations between cumulative BM dose and sex, age, SES and medical history among controls (both overall and by type of control) in multivariate linear regression models. Only medical history variables were found to be associated with cumulative BM dose and were retained as possible confounders and included in the fully adjusted risk model.

We estimated the association (odds ratios (OR) and 95% confidence intervals (CI)) between lymphoma risk and cumulative medical IR dose using multivariate unconditional logistic regression, adjusting for the matching variables (sex, age, and country), SES (categories of education and of SIOPS) and medical condition (listed above). Linearity of the dose-response relation was evaluated using a Generalized Additive Model (GAM) by entering dose as a continuous variable.

Sensitivity analyses were conducted by a) restricting the analysis to only hospital or only population-based controls; b) excluding subjects reporting any history of osteoarthritis, atopic, autoimmune or infectious disease; c) restricting analyses to participants with at least one high-dose examination (kidney, bone X-ray or CT-scan); d) excluding subjects reporting gammagraphy or any additional IR procedures not included in the dose calculation; e) using a 5 year exclusion period between dose and disease; f) using dose estimates calculated under different assumptions related to the examination time period (above); g) excluding cases interviewed over one year after diagnosis to minimize a potential survival bias; h) adjusting for smoking as a potential confounder; and i) testing for homogeneity of risk between sexes. All tests were likelihood ratio based, two-sided with a significance level of 0.05. The analysis was performed using R version 3.3 [37] and STATA 14.0 [38].

### Ethics approval

Ethics approval was obtained in 1997 from the Ethics Committee of Bellvitge hospital (coordination centre) under the project name “Eurolymph II”. Additional approval (extension of the project, reanalysis of existing data) was obtained from the International Agency for Research on Cancer (IARC) ethics review board (January 2010, project number 09–34). Ethics approval was also obtained from the relevant ethics committees of the participating countries. The list of participating institutions/hospitals can be found in S3 File. The study protocol followed was in accordance with the ethical standards of the responsible committees on human experimentation and with the Helsinki Declaration. Written consent was obtained from all participants whose data were used in the present publication.

## Results

A total of 2,362 lymphoma cases and 2,465 controls were recruited into the study. The overall participation rates were 87.7% and 68.5% respectively for cases and controls (81.1% for hospital controls and 51.3% for population controls). The median time between diagnosis and interview was 40 days (Interquartile range: 15 days; 84 days). We excluded 646 participants from analysis who reported: a) a personal history of cancer; or b) immune-depression; or c) had missing values in one of the basic adjustment variables (age, education or SIOPS). We also excluded 307 participants (7.7% of controls, 6.8% of cases) for whom data were insufficient to reconstruct their doses. A total of 1,877 cases (1599 NHL; 283 HL; 411 CLL) and 1,987 controls were included in our analyses.

Table 2 presents the characteristics of the study population. Overall, there were more men than women (56.6% male). Countries with the highest number of participants were Germany (N = 1,194) and Spain (N = 933). Only 14.9% of participants had a high education level. Atopic diseases were more prevalent in population controls, autoimmune diseases and osteoarthritis in hospital controls, and infectious diseases in cases.

**Table 2:** Basic characteristics of included cases and controls, Epilymph study, 1998–2004.

	Hospital Controls n = 1,109	Population controls n = 878	Cases n = 1,887
	n (%) or median (IQ range)	n (%) or median (IQ range)	n (%) or median (IQ range)
**Sex**			
Male	618 (55.7)	490 (55.8)	1,084 (57.4)
**Age continuous (years)**	58 (44; 69)	59 (45; 67)	59 (44; 68)
**Country**			
Spain	487 (43.9)	-	446 (23.6)
Czech Republic	273 (24.6)	-	264 (14)
France	147 (13.3)	-	180 (9.5)
Germany	-	588 (67)	606 (32.1)
Italy	-	290 (33)	205 (10.9)
Ireland	202 (18.2)	-	186 (9.9)
**Education**			
Low	493 (44.5)	390 (44.4)	858 (45.5)
Medium	471 (42.5)	351 (40)	766 (40.6)
High	145 (13.1)	137 (15.6)	263 (13.9)
**Occupational SES**^a^			
First quartile	321 (28.9)	179 (20.4)	474 (25.1)
Second quartile	360 (32.5)	273 (31.1)	585 (31)
Third quartile	211 (19)	149 (17)	367 (19.4)
Fourth quartile	217 (19.6)	277 (31.5)	461 (24.4)
**Medical History**^b^			
Family history of lymphoma	32 (2.9)	25 (2.8)	74 (3.9)
Atopic disease	513 (46.3)	470 (53.5)	930 (49.3)
Autoimmune disease	82 (7.4)	36 (4.1)	94 (5)
Infectious disease	163 (14.7)	154 (17.5)	359 (19)
Osteoarthritis	322 (29)	129 (14.7)	335 (17.8)
Cardiovascular disease	7 (0.6)	11 (1.3)	16 (0.8)
Having at least one chronic disease	416 (37.5)	293 (33.4)	600 (31.8)
Sick childhood	173 (15.6)	121 (13.8)	223 (11.8)
**Lymphoma subgroup**			
Hodgkin Lymphoma			283 (15)
Non Hodgkin Lymphoma			1599 (84.7)
Non Classifiable			5 (0.3)
**Medical radiation exposure**			
Exposed subjects	1014 (91.4)	829 (94.4)	1,632 (86.4)
Bone marrow dose (mGy)	2.27 (0.8; 5.22)	3.01 (1.09; 6.58)	2.01 (0.77; 4.66)

n: numbers; IQ: interquartile range

^a^ Standard International Occupational Prestige Scale (SIOPS);

^b^ See methods for the definition of each medical history variable

The median (Interquartile range—IQ) number of examinations excluded (because of the two-year latency period) was 1 (0; 2) for thorax X-rays, 1 (0; 1) for CT-scans and 0 for all other examinations (S3 Table). A total of 86.4% of cases and 92.7% of controls reported at least one examination. Among the exposed participants, the median (IQ) cumulative BM dose was 2.25 (0.82; 5.30) mGy; the mean (standard deviation—SD) was 3.89 (6.07) mGy. The maximum dose was 70.98 mGy.

We explored associations between socio-demographic and medical history variables (independent variables) and cumulative BM dose (dependent variable) among controls (Table 3). Female controls had on average, a lower IR dose (expressed in mGy) than males (beta coefficient β = -1.77; 95% CI -2.39; -1.16). Older controls tended to have received higher doses than younger controls (for each year of increasing age, the β was 0.03; 95% CI 0.01; 0.05). Population controls had somewhat higher doses than hospital controls (β = 1.01, 95% CI 0.41; 1.63). Higher cumulative BM doses were also observed among controls reporting a history of any atopic disease (β = 1.18; 95% CI 0.59; 1.78), autoimmune disease (β = 1.65; 95% CI 0.35–2.84), infectious disease (β = 1.14; 95% CI 0.34–1.95), or osteoarthritis (β = 1.58; 95% CI 0.82; 2.34), and worse childhood health status (β = 1.61; 95% CI 0.85; 2.38). Thus reporting one of the diseases was associated with an average increase in cumulative dose of approximately 50% with respect to the median dose value in the overall study population. Results were similar when stratified by type of control (Table 3).

**Table 3:** Associations of cumulative lifetime BM dose and demographic and medical characteristics for all controls together, population controls and hospital controls.

	All controls		Population controls		Hospital controls	
	(n = 1755)		(n = 689)		(n = 1066)	
	β ^a^	95%CI	β ^a^	95% CI	β ^a^	95% CI
**Sex**						
Female	**-1.77**	**-2.39; -1.16**	**-1.71**	**-2.73; -0.69**	**-1.81**	**-2.59; -1.04**
**Age continuous**	**0.03**	**0.01; 0.05**	**0.06**	**0.03; 0.1**	0.01	-0.01; 0.04
**Countries**						
*Hospital controls*	**Ref**					
Spain					**Ref**	
Czech Republic					-0.38	-1.49; 0.73
France					-1.01	-2.28; 0.27
Ireland					-0.51	-1.62; 0.6
*Population controls*	**1.01**	**0.41; 1.63**				
Germany			**Ref**			
Italy			-0.03	-1.06; 1.01		
**Education**						
Medium	0.1	-0.67; 0.87	0.04	-1.15; 1.22	0.21	-0.82; 1.25
High	0.43	-0.67; 1.53	0.79	-0.86; 2.44	0.25	-1.24; 1.74
**SIOPS**						
Second quartile	-0.03	-0.81; 0.76	-1.2	-2.58; 0.17	0.4	-0.55; 1.35
Third quartile	0.43	-0.48; 1.33	0.28	-1.28; 1.84	0.27	-0.84; 1.38
Fourth quartile	0.43	-0.5; 1.36	-0.21	-1.66; 1.24	0.66	-0.58; 1.89
**Medical History**						
Family history of lymphoma	1.38	-0.37; 3.13	-0.03	-2.95; 2.9	**2.29**	**0.1; 4.48**
Atopic	**1.18**	**0.59; 1.78**	**2.36**	**1.37; 3.34**	0.5	-0.25; 1.26
Autoimmune	**1.65**	**0.35; 2.84**	1.35	-1.07; 3.77	**1.64**	**0.2; 3.09**
Infectious	**1.14**	**0.34; 1.95**	1.25	-0.01; 2.51	**1.13**	**0.09; 2.18**
Osteoarthritis	**1.61**	**0.85; 2.38**	0.65	-0.84; 2.14	**2.11**	**1.22; 3.01**
Cardiovascular disease	0.12	-0.55; 0.79	0.92	-0.18; 2.01	-0.46	-1.3; 0.38
Sick childhood	**1.62**	**0.8; 2.44**	**2.17**	**0.8; 3.54**	**1.41**	**0.39; 2.43**

^a^β coefficients are mutually adjusted for the remaining model variables. β coefficients are expressed in mGy

Complete case analysis: controls with any missing data in medical history variables were excluded from analysis (n = 232);

Table 4 reports estimates of lymphoma risk in relation to medical IR dose measured as a) never/ever exposed; b) categories of cumulative lifetime BM dose; and c) categories of total numbers of X-rays. Dose was modeled as a categorical variable, as the results of the GAM indicated the dose-response analysis may not be linear (p = 0.005). Graphical results of the GAM are shown in S4 File.

**Table 4:** Associations of BM IR dose and lymphoma risk (odds ratio and 95% confidence intervals) (n = 3,424).

	Controls	Cases	Basic Model		Fully-adjusted Model	
			OR	95% CI	OR	95% CI
None	133	227	**Ref**		**Ref**	
Any Procedures	1625	1439	**0.51**	**0.40; 0.64**	**0.51**	**0.41; 0.65**
**Bone Marrow dose category**^a^						
0-<1 mGy	586	659	**Ref**		**Ref**	
1-<5 mGy	710	679	**0.83**	**0.71; 0.97**	**0.84**	**0.71; 0.98**
5- <15 mGy	374	267	**0.61**	**0.50; 0.74**	**0.62**	**0.50; 0.76**
≥15 mGy	88	61	**0.60**	**0.42; 0.85**	**0.63**	**0.44; 0.9**
P for trend			**<0.001**		**<0.001**	
**Number of X-rays**						
None	133	227	**Ref**		**Ref**	
1–4 X-ray	465	464	**0.57**	**0.45; 0.74**	**0.58**	**0.45; 0.75**
5–14 X-rays	638	608	**0.54**	**0.42; 0.69**	**0.55**	**0.43; 0.7**
>15 X-rays	522	367	**0.39**	**0.30; 0.50**	**0.39**	**0.30; 0.51**
P for trend			**<0.001**		**<0.001**	

Unconditional logistic regression model. Basic model is adjusted for sex, age, country and SES variables. Fully adjusted model is adjusted also for positive medical history of atopic, autoimmune, or infectious disease or osteoarthritis and/or sick childhood. Complete case analysis: participants with any missing data in medical history variables were excluded from analysis (n = 440).

^a^ The mean dose between the first and last exposure was attributed to the “n-2” examinations. See methods for details. Mean dose value in each dose category: 0.33 mGy [0-<1 mGy]; 2.59 mGy [1-<5 mGy]; 8.31 mGy [5-<15 mGy]; 25.23 mGy [≥15 mGy]. Maximum dose in ≥15mGy category is 70.98 mGy.

Ever having had an IR diagnostic examination resulted in a reduced OR for lymphoma risk (OR = 0.50, 95% CI 0.41–0.65). Most ORs by category of cumulative BM dose were below 1.0 in comparison with the reference category (no exposure or dose less than 1 mGy), both in the crude and in the fully-adjusted models (Table 4). Similar results were obtained when using measures of the BM cumulative dose estimated under the alternative exposure assumptions (S4 Table) and using a 5-year exclusion period (Table 5). Stronger inverse associations were observed in analyses based on categories of total numbers of examinations compared to analyses by categories of doses (Table 4). Results of analysis by subtype of lymphoma (HL, NHL, lymphoma excluding CLL, and CLL only) are presented in Table 5. Overall, a similar pattern of reduced ORs was found for each lymphoma subtypes, however for HL the evidence of a decrease trend is not statistically significant.

**Table 5:** Cumulative lifetime BM dose from common radio-diagnostic examination and lymphoma risk estimates (odds ratio and 95% confidence intervals) by subtype of lymphoma, type of control, disease status, time between interview and diagnosis, type of examination, using a 5 year exclusion period and adjusting for smoking status.

BM Dose category^a^	Controls	Cases	Adjusted OR	95% CI	Controls	Cases	Adjusted OR	95% CI
	A. Hodgkin lymphoma (2009)				B. Non Hodgkin lymphoma (3168)			
0-<1 mGy	586	139	Ref		586	518	Ref	
1-<5 mGy	710	78	0.71	0.51; 0.99	710	598	0.88	0.74; 1.04
5- <15 mGy	374	27	**0.54**	**0.33; 0.87**	374	240	**0.64**	**0.51; 0.79**
≥15 mGy	88	7	0.85	0.36; 2.02	88	54	**0.62**	**0.43; 0.91**
P for trend			**0.16**				**<0.001**	
	C. All lymphoma, excluding CLL (3418)				D. Only CLL (2098)			
0-<1 mGy	586	555	Ref		586	104	Ref	
1-<5 mGy	710	522	**0.80**	**0.68; 0.95**	710	157	1.03	0.78; 1.39
5- <15 mGy	374	198	**0.58**	**0.47; 0.72**	374	69	0.77	0.53; 1.39
≥15 mGy	88	51	**0.68**	**0.46; 0.99**	88	10	0.48	0.24; 1.01
P for trend			**0.001**				**0.014**	
	E. Only population controls (1338)				F. Only hospital controls (2106)			
0-<1 mGy	200	227	Ref		386	432	Ref	
1-<5 mGy	277	243	**0.76**	**0.58; 0.99**	433	436	0.87	0.72; 1.06
5- <15 mGy	172	128	**0.63**	**0.46; 0.86**	202	139	**0.61**	**0.46; 0.79**
≥15 mGy	42	32	0.68	0.40; 1.14	46	29	0.62	0.38; 1.02
P for trend			0.05				**0.002**	
	G. Participants reporting no disease (904)^a^				H. Participants reporting at least one disease (2970)^b^			
0-<1 mGy	171	223	Ref		415	436	Ref	
1-<5 mGy	152	146	**0.73**	**0.54; 0.98**	558	533	0.87	0.73; 1.03
5- <15 mGy	61	45	**0.53**	**0.35; 0.81**	313	222	**0.63**	**0.51; 0.78**
≥15 mGy	6	8	0.98	0.33; 2.74	82	53	**0.56**	**0.40; 0.80**
P for trend			0.07				**< 0.001**	
	I. Population controls and participants reporting no disease (589) ^b^				J. Excluding cases with an interval between diagnosis and interview larger than 1 year (exclusion of 76 cases)			
0-<1 mGy	63	88	Ref		586	634	**Ref**	
1-<5 mGy	61	56	0.71	0.49; 1.03	710	654	**0.84**	**0.71; 0.98**
5- <15 mGy	26	27	0.70	0.44; 1.13	374	259	**0.63**	**0.51; 0.77**
≥15 mGy	2	5	0.92	0.34; 2.57	88	58	**0.63**	**0.44; 0.91**
P for trend			0.68				**<0.001**	
	K. Excluding participants not reporting any CT-scan, kidney or bone X-ray (2209)				L. Excluding subjects who reported gammagraphy or other examination in free text (2416)			
0-<1 mGy	195	160	Ref		461	476	Ref	
1-<5 mGy	563	511	1.07	0.83; 1.37	520	492	0.90	0.75; 1.08
5-<15 mGy	362	255	0.82	0.62; 1.08	219	160	**0.69**	**0.53; 0.89**
≥15 mGy	88	60	0.80	0.53; 1.20	55	33	**0.60**	**0.38; 0.96**
P for trend			**0.04**				**0.002**	
	M. Lymphoma risk using a 5 year lag- period				N. Adjustment for smoking status (ever/never)			
0-<1 mGy	693	737	Ref		586	659	**Ref**	
1-<5 mGy	798	680	0.88	0.75; 1.01	710	679	**0.83**	**0.70; 0.97**
5- <15 mGy	291	211	**0.66**	**0.53; 0.88**	374	267	**0.61**	**0.50; 0.74**
≥15 mGy	67	45	**0.65**	**0.43; 0.97**	88	61	**0.62**	**0.43; 0.89**
P for trend			**0.001**			**<0.001**	

Unconditional logistic regression models adjusted for matching variables, SES, family history of lymphoma and medical history positive to atopic, autoimmune, infectious, osteoarthritis and/or sick childhood. All models are fully-adjusted with the exception of model E, F, G, H, and I which are adjusted for matching variables and SES.

Participants with any missing data in medical history variables were excluded from analysis.

Dose category based on the second scenario assumption.

^a^ Mean dose value in each dose category: 0.33 mGy [0-<1 mGy]; 2.59 mGy [1-<5 mGy]; 8.31 mGy [5-<15 mGy]; 25.23 mGy [≥15 mGy]. Maximum dose in ≥15mGy category is 70.98 mGy.

^b^Disease categories are: atopic, autoimmune, osteoarthritis and infectious diseases.

Findings were similar when restricting analyses to population or to hospital controls (Table 5). When we restricted the analysis to subject who did not report any chronic diseases there was no longer evidence of a dose-related inverse trend, with the OR in the highest dose category being close to 1.0. Similar results were obtained when additionally restricting to population controls, though the sample size was small (6 cases and 8 controls with dose >15 mGy) (Table 5). In further sensitivity analyses including only subjects who reported at least one high dose examination (CT-scan, kidney or bone X-ray) the strength of the inverse association weakened and none of the categorical ORs were statistically significantly different from 1.0 (Table 5). When we excluded subjects (N = 1,094) who reported any gammagraphy or any additional IR examination as free text, results were similar to those of the main analyses (Table 5). Risk estimates did not change when excluding the 76 cases interviewed more than one year after diagnosis, to minimize a possible effect of survival bias. Adjusting for smoking status (ever/never) did not change the results and we had no evidence for heterogeneity of risk by sex (p = 0.94, not shown).

## Discussion

We estimated the cumulative IR BM dose from the most common radiological examinations reported in the large multinational Epilymph study. IR doses tended to be very low (median 2.25 mGy), of the order of one year of natural background radiation on average [39], with none of the subjects exceeding 100 mGy. We found no increased risk of lymphoma from IR exposure from common self-reported medical radiological procedures. Indeed, ORs were generally <1.0 when comparing each exposure category to the reference category (no exposure/exposure less than 1 mGy), possibly reflecting unmeasured confounding, an unidentified source of systematic bias, or chance, considering the low statistical power of the study.

### Selection bias

Selection bias arises if participation is related to the exposure of interest. Hospital controls may have been suffering from a chronic disease (and were possibly more likely to have undergone medical IR): this could explain the higher levels of exposure among controls and the reduced ORs for lymphoma risk (S5 File). However, analyses by type of controls showed similar results and we saw no substantial difference in disease prevalence between population and hospital controls. Population controls also had a higher BM dose when compared to controls recruited in the hospital. Participation rates were lower among controls compared to cases, particularly among population controls. It is possible that the controls who participated represent a population receiving a different type of medical attention (i.e. controls who were more worried about their health or with poorer general health may be more likely to participate in a medical research study) than the population from which they were drawn. Indeed, when excluding subject reporting any chronic disease, we didn’t found evidence of a decrease trend.

### Confounding

The aetiology of lymphoma is complex, and putative risk factors may not be consistently associated across all lymphoma subtypes [22]. We adjusted all our analysis for family history, which has been found to be the most consistent risk factor across all NHL subtypes [22]. Chronic diseases may also be relevant confounders as they are associated with both diagnostic IR exposure and lymphoma risk (S5 File). A negative association between lymphoma and atopic, autoimmune and osteoarthritis has been reported [22, 23] and this could explain our inverse ORs. The association between medical conditions and lymphoma risk has been evaluated in the Epilymph study [25, 40], with reduced OR observed for allergies, osteoarthritis, diabetes, and those reporting to have been “more sick than siblings during childhood”.

Subject who reported any atopic, autoimmune, or infectious disease as well as osteoarthritis had a higher average estimated BM dose, as did participants with poorer health during childhood. This higher dose is expected as such diseases may require radio-diagnostic evaluation.

Although we were unable to explore the potential impact of chronic disease in depth here, as the medical reason/indication for the diagnostic procedure was not available, we adjusted analyses for atopic, autoimmune, osteoarthritis, infectious, and childhood diseases. This did not change the risk estimates, neither did excluding subjects who reported any chronic disease (atopy, autoimmune, infectious disease or osteoarthritis). However, as information on medical history was self-reported and may be subject to recall bias, adjustment may not be adequate.

We adjusted for socioeconomic status, as it has been reported that the exposure to medical ionizing radiation may be related to the socioeconomic status [41]. Again, adjustment did not change results, however we may not fully capture the socioeconomic status of the subject, as we measured it with proxies (occupational SIOPS score, education level).

### Uncertainty in the dose estimation

For the individual dose estimation, we had limited data on lifetime radiological history, leading to uncertainties in dose estimates. First, we lacked information on the exact time period and age at each procedure. In general, doses have decreased in recent time periods, especially in younger patients as a result of increased awareness and optimization [29, 42]. To address this, we developed three exposure scenarios with different assumptions regarding the timing of examinations; sensitivity analyses using the two alternative scenarios showed that these assumptions did not influence our risk estimates.

X-ray procedures were defined in broad terms in the questionnaire. The advantage of using such broad, generic terms is to make the questions shorter and simpler. However, when reconstructing dose, it is a major source of uncertainty since doses could vary substantially depending on the anatomical region examined. Reported BM doses from the most common CT-scan type performed in adults can vary between 6.9 mGy (chest CT-scan), 3 mGy (abdomen CT-scan) and 1.3 mGy (head CT-scan) [30]. As no information on body part was specified for CT-scans, we used a weighted average considering the distribution of body part scanned in different age groups (for adults we attributed 3.32 mGy to each CT-scan). Thus, in adults we have potentially overestimated the dose for 40% of the procedures (head CT-scans) and underestimated it for 30% (chest CT-scan). However, the percentage of participants who reported a CT scan is low (18%) and the body-part uncertainty is unlikely to be differential between cases and controls.

Furthermore, our cumulative BM dose estimation does not include all types of medical radiological procedures, only the most common. Misclassification due to the omission of other radiological procedures cannot be excluded. To further address this issue, we excluded subjects who reported a previous tumor because they may have received radiotherapy. In a sensitivity analysis, we also excluded subjects reporting gammagraphy and additional procedures (reported as free text) with no change in ORs. However, differential misclassification due to omission of uncommon radiological procedures, though unlikely to affect a large proportion of the population, cannot be excluded if controls (in particular hospital controls) were more likely to have undergone procedures not considered here, due to specific underlying chronic medical conditions.

Finally, the choice of BM as the target organ may also lead to uncertainty as it may be inadequate for some lymphoma subtypes: all lymphoid cells originate in the BM, however, their differentiation occurs only partially in the BM and, in adults, forms of lymphomas that arise from peripheral cells are more frequent [43]. However, in radiation epidemiology, BM is traditionally accepted as the target organ for lymphomas and leukemia and using it allows comparisons with the published literature. Although some studies have used alternative target organ doses, for example mean lymphocyte dose in a study of pediatric CT-scans [14], we were limited by the available information in the study. [14].

### Exposure information bias

This study is based on self-reported data and it was not possible to check medical records to validate responses. The informed consent signed by the participants allowed the researchers to access medical records only in the hospital where they were recruited, and hence we are unable to validate all of the procedures reported by the patient. In addition, considering the age range of the participants, most of the procedures (in particular those performed in childhood) are likely to have been recorded in paper charts, not in electronic medical records, thus making them very difficult to access. A medical records review that cannot assess the complete lifetime diagnostic exposure of the subjects would be difficult to interpret [44].

As we based our analyses on self-reported medical radiological history, recall error (random and systematic, differential or not between cases and controls) is likely. Previous studies have attempted to estimate the impact of recall bias when collecting medical radiological history [44–46]. Dreger et al. (2015) found evidence of underreporting in older populations and of over-reporting of CT-scans possibly due to confusion with Magnetic Resonance Imaging (MRI), however comparison was only for recent procedures (not covering the entire lifetime of the study subjects), thus the measurement error in our study may be greater. Berrington et al. [44] reported substantial disagreement between medical record based and self-reported medical radiological history. Such disagreement was shown to be independent of sex, age at interview, time since exposure and calendar year at exposure. Disagreement was also non-differential between cases and controls. However, the accuracy of reporting tended to decrease with increasing number of procedures reported in medical records. The study also compared risk estimates of thyroid cancer from medical IR using information from medical records with risk estimates based on self-reported information; no substantial difference in risk estimates was observed. Similarly, Pogoda et al. [45] did not report important differences when comparing acute myeloid leukemia risk from medical diagnostic IR exposure based on medical records or self-reported.

Even when non-differential between cases and controls, recall error can affect risk estimates, though the direction of the effect is unpredictable [47]. The observed reduced ORs in our analyses may be partially explained by non-differential misclassification, if the misclassification occurs between the higher and the lower dose categories [48]. Individuals with higher dose may have been classified as non-exposed simply because they failed to report a single high dose examination (CT-scan) or if they underwent other high dose examinations that weren´t considered in the present study. An individual with no radiation exposure could also be classified as highly exposed if diagnostic procedures that do not involve IR (e.g. magnetic resonance imaging, ultrasound) were confused with X-ray procedures (CT-scan, other X-rays). Participants with chronic diseases may also have greater misclassification as they may be a sub-group that receives more “uncommon” radiological examinations (categorized as unexposed, but truly high exposed), or non-IR diagnostic examination (magnetic resonance imaging) that may be confused with CT-scans. In this sense, the analyses excluding subjects who reported the medical conditions considered should be less affected by potential non-differential misclassification. Such analysis did not shown a decrease trend.

Our findings could also be explained by a tendency of controls to over-report or a tendency among cases to under-report the number of procedures, however, such differential bias has not previously been reported in similar studies [44, 45].

### Reverse causation bias

In our analysis, we could not specifically identify examinations taken for the lymphoma diagnosis, and including them may lead to reverse causation bias. To avoid this, we excluded all examinations taken in the two years prior to diagnosis/reference date. The median number of excluded examinations in cases is in line with the number of examinations required for a lymphoma diagnosis (S3 Table), which is normally one CT-scan and one thoracic X-ray [49]. In addition, if reverse causation bias had an impact on cases, we would have expected a positive bias in ORs. Further, since analysis stratified by type of controls lead to similar results, it is unlikely that our risk estimates were affected by hospital controls possibly receiving medical X-rays during hospitalization.

### Statistical power

To our knowledge, this is the largest case-control study evaluating medical IR exposure and subsequent lymphoma risk. However, the doses estimated were very low and hence the statistical power was limited to detect the small increase in risk that might be expected, based on current radiation risk estimates, from such dose levels. In addition, the literature review approach to exposure assignment could have introduced Berkson error [50], as we attributed exposure based on average dose values by time period, likely to further reduce the statistical power of the study. Analyses by sub-type of lymphoma were based on lower numbers and hence had even lower power. In our analysis, however, the ORs for NHL, CLL, and excluding CLL all showed similar and consistent reduced risks.

Our results should be interpreted in the context of the literature of lymphoma risk after exposure to medical diagnostic IR. The low study power and low exposure level makes it difficult for our study to reach adequate conclusions.

## Conclusion

We found no positive association between categories of cumulative lifetime BM dose (or number of procedures) from common medical diagnostic procedure and risk of lymphoma. Overall the doses were very low, thus we would have expected no increased risk or at most a very small increased risk. The reduced ORs observed here may be explained by some methodological bias we have been unable to identify, to residual confounding, or to chance. Future studies will benefit from collection of additional information on the time period, body part and the reason for each examination to overcome such possible uncertainties. Information on medical conditions should also be collected together with radiological history, given their potential confounding effect. Control selection should also aim to reduce possible participation biases related to previous medical history and therefore diagnostic IR exposure.

## Supporting information

S1 Table Bone marrow dose (mGy) attributed to each conventional X-ray examinations. (DOCX)

S2 Table Bone marrow dose attributed to each CT scan according to sex, age and time period. (DOCX)

S3 Table Median and interquartile range of number of excluded examinations, included examinations and derived cumulative bone marrow dose. (DOCX)

S4 Table Associations of BM IR dose and lymphoma risk (odds ratio and 95% confidence intervals) (n = 3,424) using alternative exposure assumptions. (DOCX)

S1 File Medical radiological history section of the interview. (DOCX)

S2 File Formula to calculate the cumulative BM dose. (DOCX)

S3 File List of participating hospitals and research centers. (DOCX)

S4 File Generalized additive model showing dose response relationship for each mGy of increasing of cumulative bone marrow dose. (DOCX)

S5 File Graphical representation of selection bias and confounding. (DOCX)
