# Correction: Association of ionizing radiation dose from common medical diagnostic procedures and lymphoma risk in the Epilymph case-control study

**DOI:** 10.1371/journal.pone.0243396

**Published:** 2020-12-01

**Authors:** Elisa Pasqual, Michelle C Turner, Esther Gracia-Lavedan, Delphine Casabonne, Yolanda Benavente, Isabelle Thierry-Chef, Marc Maynadié, Pierluigi Cocco, Anthony Staines, Lenka Foretova, Alexandra Nieters, Paolo Boffetta, Paul Brennan, Elisabeth Cardis, Silvia de Sanjose

The sixth author's name is missing a hyphen. The correct name is: Isabelle Thierry-Chef. The correct citation is: Pasqual E, Turner MC, Gracia-Lavedan E, Casabonne D, Benavente Y, Thierry-Chef I, et al. (2020) Association of ionizing radiation dose from common medical diagnostic procedures and lymphoma risk in the Epilymph case-control study. PLoS ONE 15(7): e0235658. https://doi.org/10.1371/journal.pone.0235658.
