# Supplementary material for: Association of ionizing radiation dose from common medical diagnostic procedures and lymphoma risk in the Epilymph case-control study
Source: PLoS One. 2020 Jul 10;15(7):e0235658. doi: 10.1371/journal.pone.0235658 (PMC7351167; doi:10.1371/journal.pone.0235658)
Supplement: S1 Table — (DOCX) [file pone.0235658.s001.docx]

**Table S1: Bone Marrow Dose (mGy) attributed to each conventional x-ray examinations**

| **Conventional x-ray** | **1930-1939** | **1940-1949** | **1950-1959** | **1960-1969** | **1970-1979** | **1980-1989** | **1990-1999** | **2000-2010** |
| --- | --- | --- | --- | --- | --- | --- | --- | --- |
| Thorax | See (1), Table 3 for dose values | | | | | | | |
| Abdomen^a^ |  |  |  |  |  |  |  |  |
| Face- skull |  |  |  |  |  |  |  |  |
| Bone^b^ | 1.77 | 1.89 | 1.74 | 0.57 | 0.55 | 0.57 | 0.16 | 0.16 |
| Kidney with contrast^c^ | 6.5 | 6.5 | 6.5 | 1.25 | 0.95 | 1.65 | 1 | 1 |
| - a_ We sum together abdomen with and without contrast - b_ According to the % of the body part scanned over the total number of bone x-ray performed in general population, we calculated the dose as a weighted average of thoracic-cervical spine, thoracic spine, thoracic-lumbar spine, lumbar spine, lumbosacral spine, sacrum, pelvis (dose based on Melo 2016 (1)), extremities (where dose=0) (for the distribution in general population: “UNSCEAR 2000 Report-Vol II, Annex D” 2000 (2), Table 13 , Average value between Health Care level I Country). - c _ This examination is basically an abdominal x-ray, but different films are taken on average for each examination (two conventional abdomen projections plus images at different times depending on the contrast washout). We found that on average 10 films are taken (3), 5 for each projection (anteroposterior, lateral). We therefore multiply by 5 the dose from the abdomen x-ray as reported by Melo 2016 (1). | | | | | | | | |

1. Melo DR, Miller DL, Chang L, Moroz B, Linet MS, Simon SL. Organ Doses From Diagnostic Medical Radiography-Trends Over Eight Decades (1930 to 2010). Health Phys. 2016 Sep;111(3):235–55.

2. United Nations, editor. Sources and effects of ionizing radiation: United Nations Scientific Committee on the Effects of Atomic Radiation: UNSCEAR 2000 report to the General Assembly, with scientific annexes. New York: United Nations; 2000. 2 p.

3. Contento G, Malisan MR, Padovani R, Maccia C, Wall BF, Shrimpton PC. A comparison of diagnostic radiology practice and patient exposure in Britain, France and Italy. Br J Radiol. 1988 Feb;61(722):143–52.
