# Supplementary material for: Association of ionizing radiation dose from common medical diagnostic procedures and lymphoma risk in the Epilymph case-control study
Source: PLoS One. 2020 Jul 10;15(7):e0235658. doi: 10.1371/journal.pone.0235658 (PMC7351167; doi:10.1371/journal.pone.0235658)
Supplement: S2 Table — (DOCX) [file pone.0235658.s002.docx]

**S2 Table: Bone Marrow Dose attributed to each CT scan according to sex, age and time period**

|  | **male** | | | | | **female** | | | | |
| --- | --- | --- | --- | --- | --- | --- | --- | --- | --- | --- |
| **age** | **0-4** | **5-9** | **10-14** | **15-19** | **>20** | **0-4** | **5-9** | **10-14** | **15-19** | **>20** |
| **CT-scan before 2001^a^** | 6.37 | 6.68 | 4.84 | 3.73 | 2.53 | 6.37 | 6.80 | 4.84 | 5.03 | 3.01 |
| **after 2001^a^** | NA | NA | NA | NA | 2.16 | NA | NA | NA | NA | 2.63 |
| NA (Not Applicable, we do not have participants in that age group for that time period)  a_ Dose values based on dose values reported by Kim et al (1). According to the % of the body part (chest, brain, abdomen, extremities) scanned over the total number of CT scan performed in general population, we calculated the dose as a weighted average of chest, brain, abdomen and extremities (where dose=0) CT-scan (for the distribution in general population we used for children (2) and for adults(3). | | | | | | | | | | |

References

1. Kim KP, Berrington de Gonzalez A, Pearce MS, Salotti JA, Parker L, McHugh K, et al. Development of a database of organ doses for paediatric and young adult CT scans in the United Kingdom. Radiat Prot Dosimetry. 2012 Jul 1;150(4):415–26.

2. Bosch de Basea M, Salotti JA, Pearce MS, Muchart J, Riera L, Barber I, et al. Trends and patterns in the use of computed tomography in children and young adults in Catalonia - results from the EPI-CT study. Pediatr Radiol. 2016 Jan;46(1):119–29.

3. Cruces RR, Sergio Cañete Hidalgo, Manuel Perez Martinez, Aurora Pola Gallego de Guzmán, Sonia Moreno Corrales, María Isabel Fernández Vázquez. DOPOES Report [Internet]. 2014 [cited 2018 Jan 4]. Available from: https://www.csn.es/documents/10182/1006281/Informe%20sobre%20los%20procedimientos%20de%20radiodiagn%C3%B3stico%20m%C3%A9dico%20utilizados%20en%20los%20centros%20sanitarios%20espa%C3%B1oles,%20su%20frecuencia%20y%20las%20dosis%20recibidas%20por%20los%20pacientes%20y%20la%20poblaci%C3%B3n%20(DOPOES)
