# Supplementary material for: Association of ionizing radiation dose from common medical diagnostic procedures and lymphoma risk in the Epilymph case-control study
Source: PLoS One. 2020 Jul 10;15(7):e0235658. doi: 10.1371/journal.pone.0235658 (PMC7351167; doi:10.1371/journal.pone.0235658)
Supplement: S3 Table — (DOCX) [file pone.0235658.s003.docx]

**S3 Table: Median and interquartile range of number of excluded examinations, included examinations and derived cumulative bone marrow dose.**

|  | **Number of excluded examinations** | | | | **Number of included examinations** | | | | **Cumulative BM dose of the included examinations** | | | |
| --- | --- | --- | --- | --- | --- | --- | --- | --- | --- | --- | --- | --- |
|  | p 25 | median | p75 | max | p 25 | median | p75 | max | p 25 | median | p75 | max |
| Thorax x-ray | 0 | 1 | 2 | 40 | 1 | 4 | 9 | 129 | 0.1 | 0.4 | 1.0 | 16.8 |
| CT-scan | 0 | 1 | 1 | 20 | 0 | 0 | 0 | 21 | 0 | 0 | 0 | 61.8 |
| Abdomen x-ray | 0 | 0 | 2 | 18 | 0 | 0 | 1 | 81 | 0 | 0 | 0.2 | 27 |
| Bone x-ray | 0 | 0 | 2 | 35 | 0 | 1 | 4 | 100 | 0 | 0.3 | 1.5 | 57.8 |
| Kidney x-ray | 0 | 0 | 1 | 6 | 0 | 0 | 0 | 30 | 0 | 0 | 0 | 40.8 |
| Face x-ray | 0 | 0 | 1 | 6 | 0 | 0 | 0 | 28 | 0 | 0 | 0 | 12.8 |
| Total_xray | na | na | na |  | 3 | 7 | 15 | 150 | 0.6 | 1.9 | 4.75 | 71.0 |
|  |  |  |  |  |  |  |  |  |  |  |  |  |
