# Supplementary material for: Association of ionizing radiation dose from common medical diagnostic procedures and lymphoma risk in the Epilymph case-control study
Source: PLoS One. 2020 Jul 10;15(7):e0235658. doi: 10.1371/journal.pone.0235658 (PMC7351167; doi:10.1371/journal.pone.0235658)
Supplement: S4 Table — (DOCX) [file pone.0235658.s004.docx]

**S4 Table: Associations of BM IR dose and lymphoma risk (Odds ratio and 95% Confidence intervals) (n=3,424) using alternative exposure assumptions**

|  | **Controls** | **Cases** | **Crude OR (95% CI)** | **Adjusted OR (95% CI)** |
| --- | --- | --- | --- | --- |
| **Bone Marrow dose category** |  |  | 3424 | 3424 |
| **Based on first scenario:** we attributed to the “n-2” examinations the mean dose between the first and last exposure^a^ | | | | |
| 0-<1 mGy | 586 | 659 |  |  |
| 1-<5 mGy | 710 | 679 | **0.83 (0.71; 0.97)** | **0.84 (0.71; 0.98)** |
| 5- <15 mGy | 374 | 267 | **0.61 (0.50; 0.74)** | **0.62 (0.50; 0.76)** |
| ≥15 mGy | 88 | 61 | **0.60 (0.42; 0.85)** | **0.63 (0.44; 0.9)** |
| **Second scenario estimation:** we attributed to the “n-2 examinations” the dose at age at first exposure ^a^ | | | | |
| 0-<1 mGy | 574 | 650 |  |  |
| 1-<5 mGy | 655 | 646 | **0.85 (0.72; 0.99)** | 0.85 (0.72; 1) |
| 5- <15 mGy | 407 | 283 | **0.59 (0.48; 0.71)** | **0.59 (0.48; 0.72)** |
| ≥15 mGy | 122 | 87 | **0.6 (0.44; 0.81)** | **0.64 (0.47; 0.87)** |
| **Third scenario estimation:** we attributed to the “n-2” examinations the dose at age at last exposure ^a^ | | | | |
| 0-<1 mGy | 616 | 697 |  |  |
| 1-<5 mGy | 756 | 690 | **0.78 (0.67; 0.91)** | **0.8 (0.68; 0.94)** |
| 5- <15 mGy | 341 | 247 | **0.61 (0.5; 0.75)** | **0.62 (0.5; 0.76)** |
| ≥15 mGy | 45 | 32 | **0.62 (0.39; 0.99)** | 0.66 (0.41; 1.07) |
| Unconditional logistic regression model. Basic model is adjusted for sex, age, country and SES variables. Fully Adjusted model is adjusted also for family history of lymphoma and medical history positive to atopic, autoimmune, infectious, osteoarthritis and/or sick childhood. Participants with any missing data in medical history variables were excluded from analysis. | | | | |
| a) Result of first scenario already presented in table 4 of the manuscript. Here a reported again to facilitate comparison. For the definition of each scenario, see methods for details. | | | | |
