# Supplementary material for: Association of ionizing radiation dose from common medical diagnostic procedures and lymphoma risk in the Epilymph case-control study
Source: PLoS One. 2020 Jul 10;15(7):e0235658. doi: 10.1371/journal.pone.0235658 (PMC7351167; doi:10.1371/journal.pone.0235658)
Supplement: S1 File — (DOCX) [file pone.0235658.s005.docx]

**Supplementary File S1: Details on the questions on medical radiological history asked.**

| 4.10- It is probable that during your life you have had an X-ray. I will list you different parts of the body and of X-ray and you will indicate if you have had any of them. | | | | | | | | | | | | | | | | | |  |  |  |
| --- | --- | --- | --- | --- | --- | --- | --- | --- | --- | --- | --- | --- | --- | --- | --- | --- | --- | --- | --- | --- |
|  | **Site** | | |  | | | | | **No. Of times** | | | | **Age first** | | | | **Age last** | | | |
|  |  | | |  | | | | |  | | | |  | | | |  | | | |
|  | Teeth | | |  |  | Yes |  | No |  |  |  |  |  |  |  |  |  |  |  |  |
|  |  | | |  | | | | |  | | | |  | | | |  | | | |
|  | Thorax | | |  |  | Yes |  | No |  |  |  |  |  |  |  |  |  |  |  |  |
|  |  | | |  | | | | |  | | | |  | | | |  | | | |
|  | Abdomen with no contrast | | |  |  | Yes |  | No |  |  |  |  |  |  |  |  |  |  |  |  |
|  |  | | |  | | | | |  | | | |  | | | |  | | | |
|  | Bones | | |  |  | Yes |  | No |  |  |  |  |  |  |  |  |  |  |  |  |
|  |  | | |  | | | | |  | | | |  | | | |  | | | |
|  | Face / scalp | | |  |  | Yes |  | No |  |  |  |  |  |  |  |  |  |  |  |  |
|  |  | | |  | | | | |  | | | |  | | | |  | | | |
|  | Kidneys with | | |  | | | | |  | | | |  | | | |  | | | |
|  | Contrast | | |  |  | Yes |  | No |  |  |  |  |  |  |  |  |  |  |  |  |
|  |  | | |  | | | | |  | | | |  | | | |  | | | |
|  | Abdomen with contrast | | |  | | | | |  | | | |  | | | |  | | | |
|  | or enema | | |  |  | Yes |  | No |  |  |  |  |  |  |  |  |  |  |  |  |
|  |  | | |  | | | | |  | | | |  | | | |  | | | |
|  | Mammography | | |  |  | Yes |  | No |  |  |  |  |  |  |  |  |  |  |  |  |
|  |  | | |  | | | | |  | | | |  | | | |  | | | |
|  | Scanner/TAC | | |  |  | Yes |  | No |  |  |  |  |  |  |  |  |  |  |  |  |
|  |  | | |  | | | | |  | | | |  | | | |  | | | |
|  | Gammagraphy | | |  |  | Yes |  | No |  |  |  |  |  |  |  |  |  |  |  |  |
|  |  | | |  | | | | |  | | | |  | | | |  | | | |
|  | Others (specify): | | |  | | | | |  | | | |  | | | |  | | | |
|  |  |  |  |  |  | Yes |  | No |  |  |  |  |  |  |  |  |  |  |  |  |
|  |  | | |  | | | | |  | | | |  | | | |  | | | |
