# Supplementary material for: Association of ionizing radiation dose from common medical diagnostic procedures and lymphoma risk in the Epilymph case-control study
Source: PLoS One. 2020 Jul 10;15(7):e0235658. doi: 10.1371/journal.pone.0235658 (PMC7351167; doi:10.1371/journal.pone.0235658)
Supplement: S2 File — (DOCX) [file pone.0235658.s006.docx]

**S2 File: formula to calculate the cumulative BM dose**

$$Cumulative lifetime BM dose={Cumulative BM}_{thorax x-ray}+{Cumulative BM}_{face x-ray}+{Cumulative BM}_{abdomen x-ray}+{Cumulative BM}_{kidney x-ray}+{Cumulative BM}_{bone x-ray}+{Cumulative BM}_{CT-scan}$$

Note: We sum when we have a complete radiological history (i.e.no missing in any of the examination variables) or when data on only one of the examinations was missing. To calculate the contribution to the cumulative dose from each examination we used the following formula (here presented for thorax x-ray):

$$Cumulative Bone marrow_{Thorax x-ray}=Dose Age at First_{Thorax}+ Dose Age at Last_{Thorax}+\left( \left( N_{Thorax}-2 \right)\times Mean Dose between Age First and Age Last_{Thorax} \right)$$

Note:

$N_{Thorax}$=total numbers of thorax x-ray

We repeated the same formula for each type of examination. This formula is for scenario 2, scenarios 1 and 3 have been calculated via substituting the “Mean dose between Age at first-age at last” with “Dose at age at first” and “Dose at age at last”, respectively.
