# Supplementary material for: Association of ionizing radiation dose from common medical diagnostic procedures and lymphoma risk in the Epilymph case-control study
Source: PLoS One. 2020 Jul 10;15(7):e0235658. doi: 10.1371/journal.pone.0235658 (PMC7351167; doi:10.1371/journal.pone.0235658)
Supplement: S3 File — (DOCX) [file pone.0235658.s007.docx]

**Supplementary file S3**

List of participating centre/hospitals:

**Czech Republic**: University Hospital Brno

**France**: Dijon (CHU du Bocage). Amiens (CHU). Montpellier (CHU, Centre Régional de Lutte contre le Cancer).

**Germany:** The study was initially approved by the ethics commission responsible for the main study centre in Heidelberg (Deutsches Krebsforschungszentrum) and subsequently confirmed by the respective commissions in the other study areas Ludwigshafen, Heidelberg, Würzburg, Hamburg, Bielefeld, München.

**Ireland**: Dublin City University, Beaumont hospital, Mater hospital, St. James's hospital, St. Vincent's Hospital, Tallaght hospital

**Italy:**  University of Cagliari Occupational health section, Businco Oncology Hospital, Cagliari, San Francesco Hospital, Cagliari.

**Spain:** ICO-L’Hospitalet, L’Hospitalet de Llobregat , Barcelona; Hospital Universitari de Bellvitge, L’Hospitalet de Llobregat , Barcelona; Hospital de Tortosa Verge de la Cinta, Tortosa, Tarragona; Hospital Universitari de Tarragona Joan XXIII, Tarragona; Hospital Universitario Ramon y Cajal, Madrid
