# Supplementary material for: Association of ionizing radiation dose from common medical diagnostic procedures and lymphoma risk in the Epilymph case-control study
Source: PLoS One. 2020 Jul 10;15(7):e0235658. doi: 10.1371/journal.pone.0235658 (PMC7351167; doi:10.1371/journal.pone.0235658)
Supplement: S4 File — (DOCX) [file pone.0235658.s008.docx]

**Supplements file S4**

**Generalized Additive Model showing dose response relationship for each mGy of increasing of cumulative bone marrow dose**

Figure 1: Generalized Additive Model showing dose response relationship for each mGy of increasing of cumulative bone marrow dose. Model adjusted for sex, age, country, education level and SIOP score.

Figure 2: Generalized Additive Model showing dose response relationship for each mGy of increasing of cumulative bone marrow dose including only population controls. Model adjusted for sex, age, country, education level and SIOP score.

Figure 3: Generalized Additive Model showing dose response relationship for each mGy of increasing of cumulative bone marrow dose including only hospital controls. Model adjusted for sex, age, country, education level and SIOP score.
