# Supplementary material for: Association of ionizing radiation dose from common medical diagnostic procedures and lymphoma risk in the Epilymph case-control study
Source: PLoS One. 2020 Jul 10;15(7):e0235658. doi: 10.1371/journal.pone.0235658 (PMC7351167; doi:10.1371/journal.pone.0235658)
Supplement: S5 File — (DOCX) [file pone.0235658.s009.docx]

**File S5: Graphical representation of selection bias and confounding**

Figure A represents the selection bias that may have biased our results: there is an unknown factor, or factors, (U) that increases or decreases the probability of taking part and these are also related to the exposure.

Figure B represent the possible confounding bias hypothesized in this study. The presence of a medical condition (MC) is associated with lymphoma risk (L) and is also associated with radiation exposure (ME), thus creating a bias pathway between ME and Lymphoma risk

Figure A

Figure B
